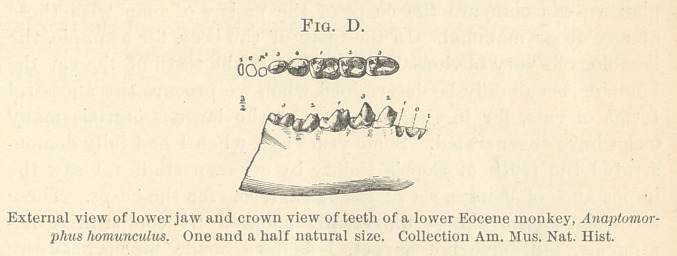# The History of the Cusps of the Human Molar Teeth

**Published:** 1895-07

**Authors:** Henry Fairfield Osborn


### International Dental Journal.

Vol. XVI. July, 1895. No. 7.

### Original Communications.[1]

### THE HISTORY OF THE CUSPS OF THE HUMAN MOLAR TEETH.[2]

BY HENRY FAIRFIELD OSBORN.[3]

Mr. President and Gentlemen,—I wish to congratulate the members present upon the formation of this *Institute of Stomatology*. It seems to me to mark one of the stages in the remarkable specialization of human knowledge when, at the present time, it is proposed to devote the work of an entire society to the scientific study of the mouth parts, as I understand your object to be; and I also gather from the fact that you have asked me, as a comparative anatomist, to deliver an address this evening, that you look at the subject in two ways,—from the stand-point of applied or practical science and from the stand-point of theory. It is on the theoretical side that I should like to bring before you this evening the *history or origin of the cusps of the human molar teeth.*

We take up this skull of an Eskimo, and you will observe that the teeth are slightly worn, and that the molars have four cusps.[4] Half a century ago this would have been considered as

[1] The editor and publishers are not responsible for the views of authors of papers published in this department, nor for any claim to novelty, or otherwise, that may be made by them. No papers will be received for this department that have appeared in any other journal published in the country.

[2] Address before the New York Institute of Stomatology, April 19, 1895.

[3] Da Costa Professor of Biology, Columbia College, New York.

[4] E. D. Cope, "On the Tritubercular Molar in Human Dentition." Journ. of Morphology, July, 1888, p. 7.

something ultimate, simply as an adaptation to human diet; but now that we have come to understand the doctrine of evolution, we ask ourselves, What is the meaning of these cusps? what is their history? what is their origin? Now, these four cusps which are present on the four corners of the teeth might be explained by evolution in three ways. We might imagine that the crown of the tooth was originally a low rounded summit, and that on the summit these four cusps appeared at each angle; no one has advocated this. Or we might imagine that they represent the coming together of a number of tips of pointed teeth, such as we see in the jaw of this member of the dolphin family; this is the theory which has been recently advanced in Germany, and it has been called the "*cusp concrescence*" theory. Or, again, we might imagine that these cusps have originated by a gradual addition to the sides of a primitive single cone; this we call the "*cusp differentiation*" theory, or the theory of cusp addition, in distinction from concrescence. The differentiation theory is supported by Cope, by myself, and others in this country.

Now, suppose an evolutionist were to trace back the history of the monkeys and of other animals among their fossil ancestors, he would find that the further back his researches extended the more simple the types of the teeth would be; he would find that the teeth of the oldest types of ancestral mammals have a simple conical form; the form that is preserved in the teeth of the whales and the dolphins of the present day, or in the Edentates, as represented in the group to which the sloth and the armadillo of South America and South Africa belong. (Fig. A.)

Fig. A.

Section of the upper and lower jaws of a dolphin, showing the alternation of simple conical teeth of the reptilian type.

We have the same type of conical tooth preserved in the human canines, and if we turn from the teeth of man, in which the canine has almost entirely lost its original laniariform or flesh-tearing shape, to that of the lower monkeys, we see that the canine is really a pointed tooth; so that we may draw a suggestion from this fact that all the teeth of the series at one time were pointed.

It is moreover true that wherever we find these pointed teeth they are present in the jaw in large numbers, sometimes sixty or seventy on one side and usually running far back into the mouth, and it is this fact which led to the suggestion of the theory of "concrescence" in the formation of molar teeth.

THE CONCRESCENCE THEORY.

You might at this stage be not inclined to take this "concrescence theory" seriously, but my address has been suggested largely by the fact that it has been taken very seriously by some well-known anatomists in Germany; as seen in the position of Professor Schwalbe,[1] in a recent article, in which he reviews the entire literature in regard to the formation of teeth published during the past fourteen or fifteen years, and concludes that in the concrescence theory and the differentiation or cusp addition theory the evidence is so evenly balanced that he cannot decide between them. It is, therefore, a question *sub judice*, and worthy of the attention of odontologists. As to the source of this theory, it was proposed simultaneously by two Germans, both of whom claim the credit of originating it. One is Dr. Carl Röse, a physician of Freiburg, a man of fine powers of research and great energy, since he has, during the past few years, issued in rapid succession a series of valuable papers on the embryological development of the teeth, which place him in the front rank of students of this subject in this decade. The other is Professor W. Kükenthal, of Jena, whose views sprang principally from the study of the teeth of whales. While these two writers are in doubt as to which should enjoy the precedence, I find, in correspondence with my friend Dr. Ameghino, of the Argentine Republic, also originally a physician and now a distinguished palæontologist, that he promulgated this theory as far back as 1880. In a work which he published at that time, entitled "Filogenia," he says, "For the reasons we are about to give it is evident that all mammals which possess compound teeth have in past periods possessed a very much larger number of teeth, but of quite simple conical form, like those of the modern dolphin. The most primitive mammals must also have had a number of very elevated teeth, but it is difficult at the present time to determine how large this number was. Nevertheless, if we take as an example a mammal in which the dentition is

---

[1] "Ueber Theorien der Dentition." Anatomischer Anzeiger Centralblatt, 1894.

complete, as in the *Macrauchenia*[1] or in the horse, and if we reduce the number of its compound teeth, we find that the most remote ancestors of these forms must have possessed more than one hundred and fifty teeth. This number is certainly not exaggerated, because *Priodon*, the giant tatusia, a mammal in an already quite advanced stage of evolution, possesses nearly one hundred simple teeth, and in the dolphin this number rises from one hundred and fifty to one hundred and seventy." I read this to show that if there is any truth in the concrescence theory, Dr. Ameghino partly deserves the credit for it. Moreover, we learn from Schwalbe that the same theory was advanced by Professor Gaudry in 1878, and still earlier by Professor Magitot in 1877.

Now let me illustrate, in a very simple manner, what is meant by the theory of concrescence and how we can imagine that the human molars have been built up by bringing together a number of isolated teeth. Placing a number of conical teeth in line, as they lie in the jaw of the whale, they would represent the primitive dentition. In the course of time a number of these teeth would become clustered together in such a manner as to form the four cusps of a human molar, each one of the whale-tooth points taking the place of one of the cusps of the mammalian tooth,—in other words, by a concrescence, four teeth would be brought into one so as to constitute the four cusps of the molar crown. Vertically succeeding teeth might also be grouped.

Fig. B.

Fragmentary lower jaw of a Jurassic mammal, *Amphilestes*, showing five triconodont molars, with three cusps in line. Greatly enlarged.

Now, what evidence is there in favor of this theory, and what is there against it? First, there is this, that all primitive types of

[1] This is one of the peculiar extinct South American hoofed animals.

reptiles from which the mammalians have descended and many existing mammals, as we have noted, have a large number of isolated teeth of a conical form; secondly, we find that by a shortening of the jaw, the dental fold or embryonic fold, from which each of the numerous tooth-caps is budded off in the course of development, may be supposed to have been brought together in such a manner that cusps which were originally stretched out in a line would be brought together so as to form groups of a variable number of cusps according to the more or less complex pattern of the crown.

What may be advanced against this theory? This, and it is conclusive to my mind: we find at the present time that cusps, quite similar in all respects to each of the cusps which form the angles of the human molar, are even now being added to the teeth in certain types of animals, such as the elephant, whose molar teeth cusps are being complicated now or until very recent times. Then we find in the mesozoic period certain animals with tricuspid teeth. Now, according to the theory of concrescence these teeth ought not to show any increase of cusps in later geological periods; but as we come through the ages nearer to the present time we find that the successors of those animals show a very much larger number of cusps. How is this increase of cusps to be accounted for? Has there been a reserve store of conical teeth to increase the cluster? No. Most obviously, to every student of the fossil history of cusps there is no reserve store, but new cusps are constantly rising up on the original crown itself by cusp addition.

However, do not let me give you the impression that these researches of Röse and Kükenthal are not of the greatest value and interest: we shall see later on how the very facts of embryology which are advanced by Dr. Carl Röse in support of his hypothesis can be turned against him and used to support the differentiation theory.

### THE DIFFERENTIATION THEORY.

Now let us turn to the differentiation theory and see what evidence we have of that. Let us go back to a very remote period of time, through the geological ages of the Pliocene and the Miocene, through the Eocene, through the Cretaceous or chalk period, and even the Jurassic. Still further back we go to the Triassic, and the interval between this and the present period has been estimated at over ten million years. Now, in the Triassic we find the mammalia, or the first animals which we can recognize as mammalia, possess conical, round, reptilian, or dolphin-like teeth. There are also some aberrant types which possess complex or multitubercular teeth.

KEY TO PLATE AA.

These teeth begin to show the first traces of cusp addition, as shown in the plate at the beginning of this article and in the accompanying key to this plate.

Here (Fig. 1) we have represented the teeth of the *Dromatherium*, an animal found in this country in the coal-beds of North Carolina, and on the sides of the main cone are cusps or rudimentary cuspules. In this enlarged model you see that on either side of the main cone are two cuspules. These teeth were found six hundred feet below the surface in a coal-mine, and in the same mine we find another animal, represented by a single tooth here (Fig. 2), in which these cusps are slightly larger. These cusps have obviously been added to the side of the tooth, and are now growing. Then we pass to teeth of the Jurassic period, found in large numbers both in America and in England, but still of very minute size; and we observe the same three cusps, but these cusps have now taken two different positions; in one case they have the arrangement represented in Fig. B, page 392: the middle cusp is relatively lower, and the lateral cusps are relatively higher; in fact, these cones are almost equal in size; these teeth are termed *triconodont*, as having three nearly equal cones. But associated with this of *Triconodont* is another animal named *Spalacotherium*, the teeth type of which are represented in Fig. 4. This is one of the most significant teeth which we have among all the fossil series, because this tooth illustrates the step that was taken in the transformation of a tooth (triconodont) with three cusps in line to a tooth with three cusps forming a *triangle;* for the primitive cusp is now seen to be the apex of a triangle, of which the two lateral cusps are the base. Now, this fact in itself is of great significance, because this tooth in this single genus is the key of comparison of the teeth of all mammalia of the great class to which man belongs. By this we are able, as you shall see, to determine that part of a human molar which corresponds with a conical reptilian tooth.

The stage shown you is the triangle stage; the next stage is the development of a heel or spur upon this triangle, as you see in Fig. 5, *Amphitherium.* To sum up: we have a reptilian cone, two cusps added to it, and a heel,—four cusps altogether, and we shall now see what relation these bear to the human molar.

First let us turn to some transitional forms. Examine a molar of the living opossum, a marsupial, which still distinctly preserves the ancient triangle. Look at it in profile, in side, or in top view, and see that the anterior part of that tooth is unmodified. This triangle we also trace through a number of intermediate types.

In this figure (Fig. 6) of *Miacis*, a primitive carnivore, we observe a high triangle and a heel, and when we come to look at it from above (6a) we find that the heel has spread out broader, so that it is as broad as the triangle. Now, the three molars of this animal illustrate a most important principle,—namely, that the anterior triangular portion of the crown has been simply levelled down to the posterior portion of the crown. Compare these three teeth, therefore, and you see illustrated a series of intermediate steps between a most ancient molar and the modern molar of the human type. The second tooth is half-way between the first and third. Look at the second molar from above and you see it has exactly the same cusps as the first, so it is not difficult to recognize that each cusp has been directly derived from its fellow. Now direct attention to the third tooth of the series (Fig. 7), for it is of equal significance with the others. This tooth has lost one of its cusps: it has lost a cusp of the triangle. It is now a tooth with only half the triangle left on the anterior side, and with a very long heel. That tooth has exactly the same pattern as the lower human molar tooth (Fig. 8); the only difference is that the heel is somewhat more prolonged. These teeth belong to one of the oldest fossil monkeys, *Anaptomorphus.*

I have no doubt many of you have observed, in the examination of human lower molars, that occasionally instead of having four cusps they have five. The fifth cusp always appears in the middle of the heel, does it not, or between the posterior lingual and the posterior buccal? You find this in the monkeys and in many other mammals, but I know of no record of the ancient anterior lingual reappearing.

So we see that the human lower molar tooth with its low, quadritubercular crown has evolved by addition of cusps and by gradual modelling from a high-crowned, simple-pointed tooth. Now this, and I say it with great confidence, is what has actually taken place. It has not come about by bringing together single reptilian cones; it has been simply by the addition of one cusp after another to an original single reptilian cone until there were six cusps, and then, in the adaptation and fitting of the lower teeth to the upper, one of the cusps has disappeared This cusp was the primitive anterior lingual, or, in comparative anatomy, the *paraconid.*

Now let us follow the history of the upper teeth and see why the "primitive anterior lingual," or paraconid, in the lower jaw has disappeared.

You are constantly, in your practice, observing that one tooth in the lower jaw gets into the way of another tooth and has to be pushed out of place in order to place its opponent in the upper jaw into its proper position. This is exactly what Nature has done; Nature has abandoned that lower cusp simply because, in the simultaneous transformation of the upper teeth from a three-cusp to a four-cusp type, there was no room for it.

### MECHANICAL RELATIONS OF THE UPPER AND LOWER TEETH.

Let us examine the upper teeth. We must say, in the first place, that our evidence here is not nearly so complete, because a lower jaw, from its thin nature, is more apt to be preserved fossil than an upper jaw; so that in the older rocks we meet with ten lower jaws to one upper jaw, and we cannot get the same evidence as to the history of the upper jaw that we have of the lower; but although we are not able to trace the history of the upper teeth with the same accuracy or degree of certainty, we have every reason to think it was the same. We find the upper teeth shaped like a *triangle*, as in Fig. 9, so we may imagine that the same triangle which was formed in the lower jaw was formed in the upper jaw, with this important difference, that in the upper jaw the base of the triangle was turned outward, whereas in the lower jaw the base of the triangle was turned inward.

What I mean by this is illustrated in the accompanying Plate BB, Figs. *A—J*, which are an epitome of the whole history. The upper teeth are represented solid, the lower teeth as hollow circles.

In *A* we see a row of single cusps, the lower somewhat inside of the upper. In *B* the lateral cusps are added. In *C* they are enlarged. In *D* the cusps are pushed outward and inward into triangles. In *E* a spur is added on the lower molar triangle, which in *F* and *G* grows out into a broad heel. In *H* and *I* a spur appears upon the upper molar triangle, and in *J* this causes the lower molar triangle to lose its anterior cusp. Nature has corrected any possible interference between these triangles in a simple manner by turning the base of the triangle of the upper molars outward towards what you call the buccal side. In the lower jaw, on the other hand, the base of the triangle is turned inward to the lingual side, so that finally we have the two triangles alternating, coming together as in *D* and making a beautiful cutting mechanism; because if any food gets in between these triangular shears the food tends to press these teeth forward and backward, therefore crowding the teeth more closely together and tending to tighten and improve the shear, whereas if the teeth were placed in line, as in *C*, and food were to get in between, the effect would be to crowd the two jaws apart and lessen the exact cutting power of the shear.

Fig. C.

Lower teeth in the left jaw of a seal, *Leptonyx leopardinus*, showing five triconodont premolars and molars, with three cusps in line.

Now we see that we can compare the lower and upper triangles to each other. How about the heels or spurs, and why were they developed? They were developed because these animals required crushers as well as cutters; they required to break up their food, and consequently a crushing surface was developed in each heel. In the course of time the animal gave up its cutting and tearing function, and in all the group of animals to which man belongs it acquired a purely crushing function, as seen in the teeth of the baboon. As that became necessary, the next step was to transform the entire upper tooth into a crusher as well as the lower, and to fill out all the spaces between them, so that a square lower tooth would abut against a square upper tooth, as in *J*, and this was done by simply adding a heel to this tooth. Now, what would that heel come against in *I?* It would come against the anterior cusp of the lower triangle; therefore that cusp had to be removed, so when the upper heel was developed this lower cusp was removed and the lower molar, which had six cusps, presented only five; then the second lingual cusp was pushed forward, as in *J*, and the tooth was transformed into a quadritubercular molar.

### EVIDENCE THAT THE UPPER HUMAN MOLARS WERE TRIANGULAR.

How do we know that is so? We have some conclusive evidence of it in other animals of the group to which man belongs. Beginning with the lemurs, the lowest type of monkeys, and entirely separate in many respects from the higher types, we find almost without exception that the upper teeth are triangular, there being no posterior cusp, so that Fig. 9, Plate AA, accurately

Fig. D.

External view of lower jaw and crown view of teeth of a lower Eocene monkey, *Anaptomorphus homunculus.* One and a half natural size. Collection Am. Mus. Nat. Hist.

represents a tooth of the lemurs, and it also represents the tooth of the true monkeys which we find in the Eocene period; in other words, all monkeys or all primates of the group to which man belongs had at the outset this triangular upper molar. Then earlier or later in the Eocene or Miocene the spur began to be developed which transformed a three-cusp tooth or a triangular tooth into a quadritubercular tooth. That spur became enlarged and finally, in civilized races of men, we have a tooth of this form as the prevailing type of tooth. These stages are shown in Plate AA, Figs. 9, 10, 11, 12.

Now, we might say that the evidence is not perfectly satisfactory, because we have no positive reason for believing that the human teeth were derived from such a type as this; they may have come along another line of descent, and for that reason we have to show here, through the kindness of one of the members of the dental profession in this city, the teeth of an Eskimo, which, as Professor Cope has pointed out, differ from the teeth of all negroes, all Indians, and all the lower races of men, in presenting in a much clearer manner the primitive triangular arrangement of the cusps that characterize the lemurs. A friend has just been telling us what very few of us knew,—that the Eskimos do not chew their food: they simply swallow it whole or gulp it down; and their food consists largely of blubber. Blubber does not form much resistance to the teeth, and, whether as a mechanical or an inherited effect of the lack of resistance of soft food through many generations of blubber-eating Eskimos or not, the teeth of these Eskimos are exceptionally *tritubercular*. This fact was pointed out by Professor Cope in his article entitled, "Lemurine Reversion in Human Dentition."[1]

Up to a certain point in their evolution the molar teeth of all mammals followed exactly the same route. It follows that if we once grasp the principles of cusp addition upon this triangular ground plan we can compare the cusps of the molars of man with those of any other mammal. In the teeth of the bear, for example, the homology is very obvious indeed. But in the teeth of the cat the homologies can only be determined when we procure the ancestral forms of cats, for in the evolution of the large sectorials many cusps have degenerated. Some years ago, when I had fully demonstrated the truth of Cope's theory by my own studies, I saw the importance of using a set of standard terms for the cusps. These have since been almost universally adopted by comparative anatomists, but have not as yet, I believe, made much headway among human odontologists. They are as follows, as applied to the human teeth:

UPPER MOLARS.

| | | |
|---|---|---|
| Anterior palatal | Protocone | Primitive triangle, or "trigon." |
| Anterior buccal | Paracone | |
| Posterior buccal | Metacone | |
| Posterior palatal | Hypocone | Primitive heel, or "talon." |

[1] Journal of Morphology.

LOWER MOLARS.

| | | |
|---|---|---|
| Anterior buccal . . . . . . | Protoconid | Primitive triangle, or "trigonid." |
| Anterior lingual . . . . . | Metaconid | |
| Posterior buccal . . . . . . | Hypoconid | Primitive heel, or "talonid." |
| Posterior lingual . . . . . | Entoconid | |
| Posterior mesial . . . . . . | Hypoconulid | |

When we understand that all the teeth of all mammals have this key, this tritubercular key, we can unlock the comparisons through the series and point out the homologies.

There is further evidence in support of the theory of cusp addition which I will now briefly mention. It is that brought forth by the very investigations of Dr. Carl Röse, which he has used to support the concrescence theory. We should expect, in the embryonic jaw that the calcification of the tooth-germ would be very significant, because we know that the embryonic structures in their development follow the order of addition or evolution. The order of evolution is, to a certain extent, repeated in embryonic development. How is it with the teeth? Dr. Röse has given a most exact account of the mode of calcification of the tooth-germ within the jaw; this is also now to be had in the form of wax models, prepared by Professor Zeigler, of Freiburg.

To begin with the lower molars, the dental cap in the jaw forms a broad, saucer-like surface, and then at the corners of that cap calcified points appear. In what order do they appear? The order is shown in the following table:

COMPARISON OF EVOLUTION AND EMBRYONIC DEVELOPMENT.

| | Order by "Cusp Addition Theory." | Order of Embryonic Development. |
|---|---|---|
| UPPER MOLARS . . . . | 1. Anterior palatal. | 1. Anterior buccal. |
| | 2. Anterior buccal. | 2. Anterior palatal. |
| | 2. Posterior buccal. | 3. Posterior buccal. |
| | 4. Posterior palatal. | 4. Posterior palatal. |
| LOWER MOLARS . . . . | 1. Anterior buccal. | 1. Anterior buccal. |
| | 2. Anterior lingual. | 2. Anterior lingual. |
| | 3. Posterior buccal. | 3. Posterior buccal. |
| | 4. Posterior lingual. | 4. Posterior lingual. |
| | 5. Posterior mesial. | 5. Posterior mesial. |

In the lower molar teeth the order of calcification is precisely the order of evolution,—in other words, the anterior buccal was the first to evolve, representing the reptilian cone; it is also the first to calcify. The anterior lingual is the second in age and also the second to calcify. The third and the fourth cusps calcify almost simultaneously. So we find that the order of embryonic development exactly repeats the order of historical development and in every way presents the strongest kind of confirmation of the theory of cusp formation which we have been discussing. But this you see is not exactly the case in the upper molars. Nevertheless, out of eight cusps in the upper and lower molars considered together, *six* cusps calcify in the order in which they were successively added to the single reptilian cone.

Gentlemen, I trust that I have not in this address taken you too far afield. I have reached a conclusion on this subject which could be elaborated in much greater detail. In closing, I would like to refer to the work of Dr. J. L. Wortman, who is here this evening, and who was for some years a collaborator with Professor Cope in Philadelphia, and who in association with Professor Cope had quite a share in the establishment of the "tritubercular or cusp addition" theory. This theory is now a rival to the "concrescence" theory; and, while it may not seem a matter of great importance, if the concrescence theory may not seem one we ought to take seriously, still, in view of the attention which it has gained in Germany, it is time that we produce and bring forward the unimpeachable evidence which we get of the history of these teeth from the rocks, the solid evidence from the geological formations, the evidence of comparative anatomy, which, as we have just seen, is so far supported by the evidence of embryonic development.

### BIBLIOGRAPHY.

Works of reference in addition to those cited above:

Röse, "Ueber die Entwickelung und Formabänderung der menschlichen Molaren." Anatomischer Anzeiger, Band vii., 1892.

Kükenthal, "Ueber den Ursprung und die Entwickelung der Säugethierzahne." Jenaische Zeitschrift für Naturwissenschaft, Band 28, 1893.

Osborn, "The Evolution of Mammalian Molars to and from the Tritubercular Type." American Naturalist, 1888, p. 1067.

"The History and Homologies of the Human Molar Cusps." Anatomischer Anzeiger, vii., 1892, pp. 740–747.

Cope, "The Mechanical Causes of the Development of the Hard Parts of the Mammalia." Journal of Morphology, iii., 1889.

## Figures and Tables

**Fig. A. f1:**
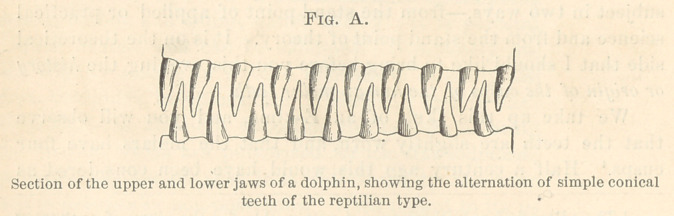


**PLATE AA. f2:**
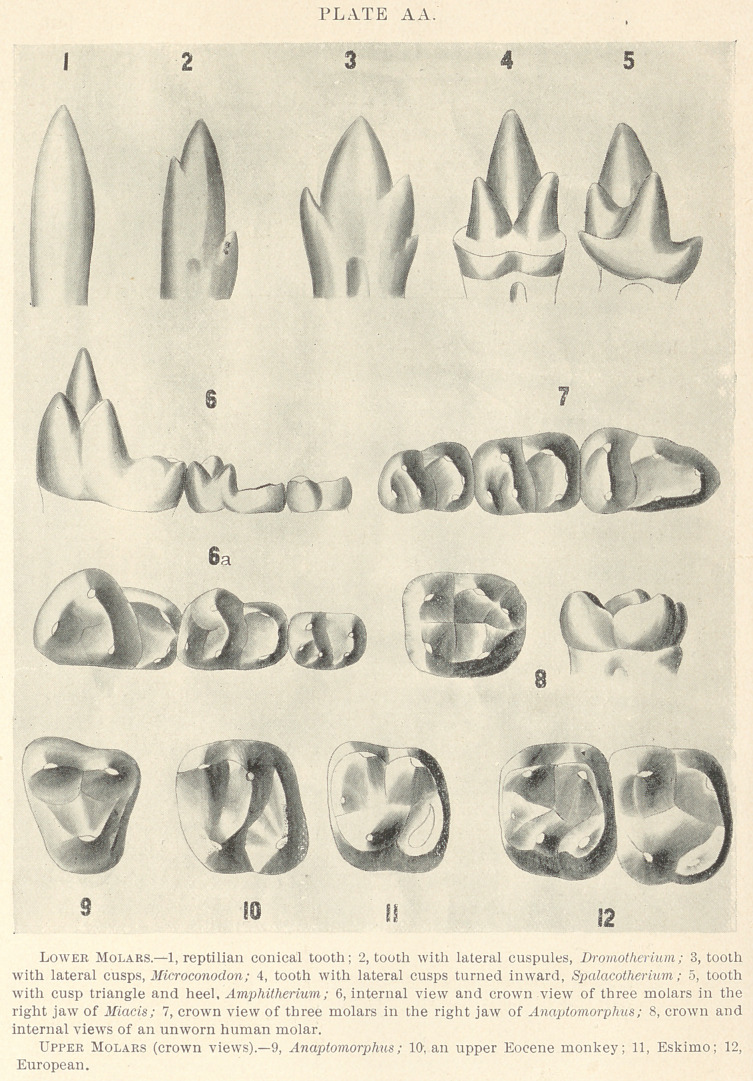


**Fig. B. f3:**
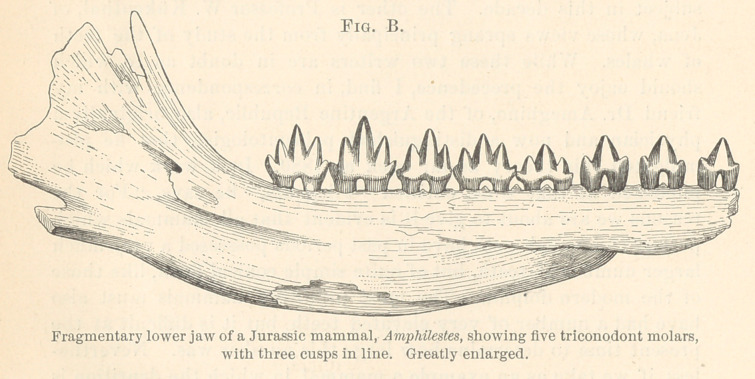


**KEY TO PLATE AA. f4:**
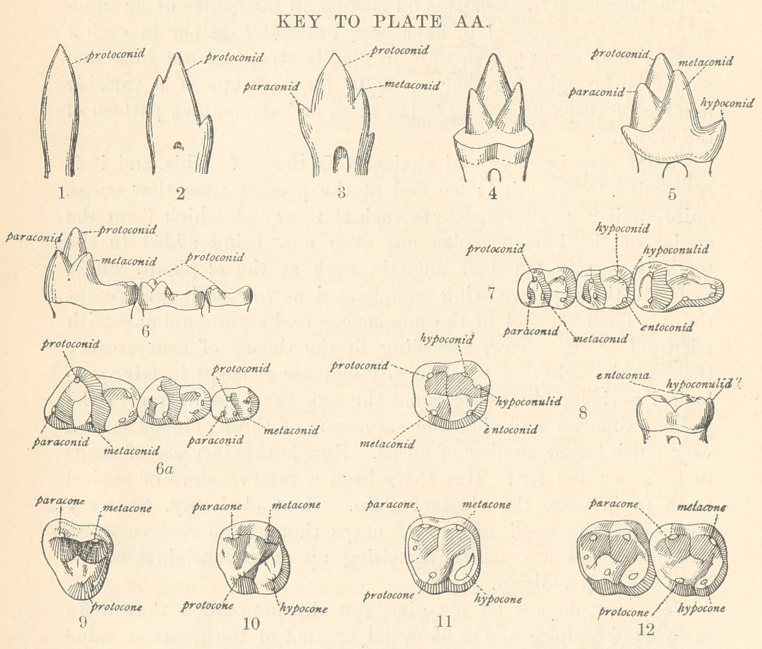


**PLATE BB. f5:**
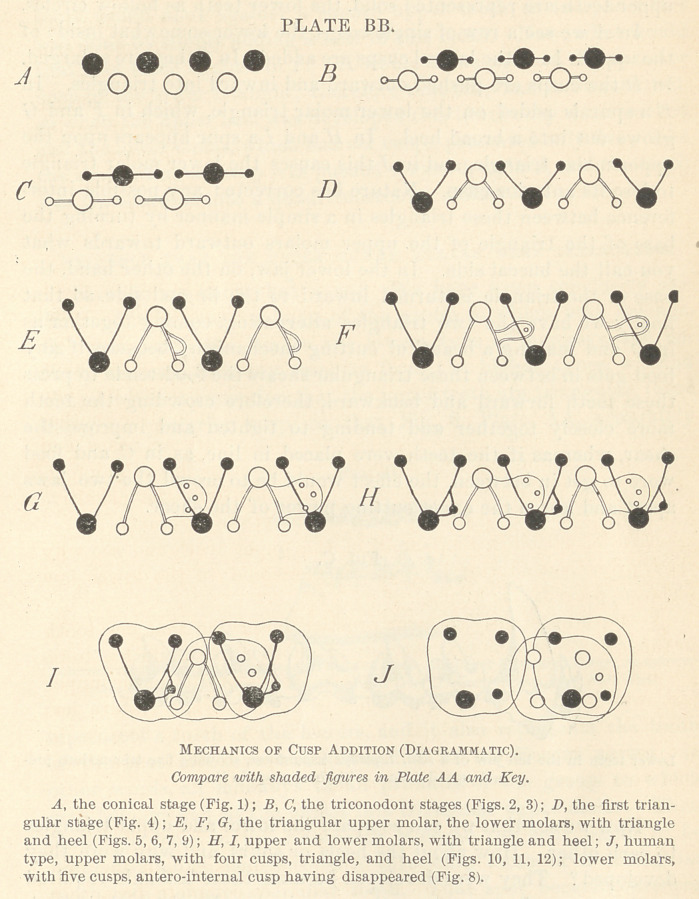


**Fig. C. f6:**
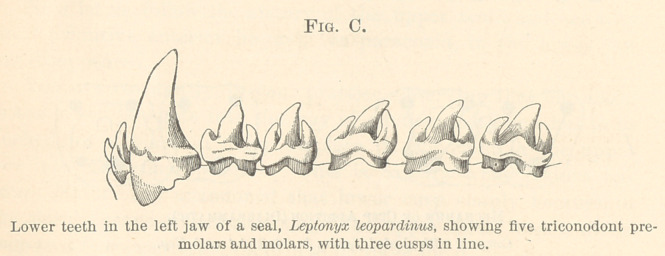


**Fig. D. f7:**